# Type 2 deiodinase p.Thr92Ala polymorphism does not affect the severity of obesity and weight loss after bariatric surgery

**DOI:** 10.1038/s41598-022-14863-x

**Published:** 2022-06-23

**Authors:** Nicoletta Benenati, Annalisa Bufano, Silvia Cantara, Claudia Ricci, Carlotta Marzocchi, Cristina Ciuoli, Ida Sannino, Andrea Tirone, Costantino Voglino, Giuseppe Vuolo, Maria Grazia Castagna

**Affiliations:** 1 Department of Medical, Surgical and Neurological Sciences, UOC Endocrinology, University of Siena, Siena, Italy; 2 Department of Surgical Sciences, Bariatric Surgery Unit, University of Siena, Siena, Italy

**Keywords:** Genetics, Endocrinology

## Abstract

A single nucleotide polymorphism in the Type 2 deiodinase (DIO2) gene (p.Thr92Ala) was found to be associated with hypertension, type 2 diabetes mellitus (T2DM), insulin resistance, and body mass index (BMI). We retrospectively evaluated 182 patients to assess whether the *DIO2* p.Thr92Ala was associated with severe obesity and response to bariatric surgery. Genomic DNA was extracted from peripheral blood leukocytes before surgery. Glycemic control parameters, cardiometabolic risk biomarkers (waist circumference, lipid assessment and blood pressure) and hormonal parameters were assessed at baseline and after surgery. Based on genotype evaluation, 78/182 (42.9%) patients were homozygous wild-type (Thr/Thr), 83/182 (45.6%) heterozygous (Thr/Ala), and 21/182 (11.5%) rare homozygous (Ala/Ala). Age at the time of the first evaluation in our Unit was significantly lower in patients with *DIO2* p.Thr92Ala. No significant association was observed between *DIO2* p.Thr92Ala and BMI, excess weight, waist circumference, Homa Index. The prevalence of comorbidities was not associated with allele distribution except for hypertension that was more frequent in wild-type patients (p = 0.03). After bariatric surgery, excess weight loss (EWL) % and remission from comorbidities occurred without differences according to genotypes. *DIO2* p.Thr92Ala does not affect the severity of obesity and its complications, but it seems to determine an earlier onset of morbid obesity. The presence of polymorphism seems not to impact on the response to bariatric surgery, both in terms of weight loss and remission of comorbidities.

## Introduction

Type 2 deiodinase (D2) is an intracellular enzyme which catalyzes the conversion of thyroxine (T4) to its active form triiodothyronine (T3) controlling the intracellular T3 concentration, its availability to the nucleus and the saturation of the nuclear T3 receptor in target tissues^1^. Therefore, D2 is a very important regulator for tissue metabolic activity. A common single nucleotide polymorphism in the *DIO2* gene, resulting in a threonine change to alanine at codon 92 (p.Thr92Ala, rs225014)^2^, has been identified in 12–36% of the general population^3^. Many works have evaluated how this polymorphism could participate as a disease mechanism and/or affect clinical outcomes^1^. The most obvious possibility is that p.Thr92Ala substitution affects D2 catalytic activity, reducing T3 production and causing either or both systemic/localized hypothyroidism^4^. Different studies have investigated the Ala92-D2 enzyme both in vitro and in vivo^5–7^. In a recent study, a comparison of presurgical hormonal status of LT4-treated thyroidectomized individuals with their post-surgery status revealed an association between low FT3 values and p.Thr92Ala variant^8^. In the carriers of the p.Thr92Ala-DIO2 polymorphism there is evidence of upregulation of pathways related to the mitochondria, Golgi apparatus/ER transport, oxidative stress, and apoptosis^9^. Population-based studies have suggested associations between p.Thr92Ala with hypertension^5,10^, bipolar disorder^11^, mental retardation^12^, low intelligence quotient (IQ)^13^, recovery from lung injury^14^, osteoarthritis^15^, increased bone turnover^16^, Graves’ and Hashimoto’s diseases^17^. In addition, many studies have shown that the p.Thr92Ala polymorphism is related to type 2 diabetes mellitus (T2DM)^16^, insulin resistance^17,18^, and body mass index (BMI)^3,4,16–21^. Interestingly, most of these associations are independent of serum thyroid hormone levels, which highlight the importance of local regulation of thyroid hormones in peripheral tissues^3^.

Thus, given the emerging essential physiological importance of DIO2, the aim of this study was to investigate the DIO2p.Thr92Ala polymorphism in relation to obesity and its comorbidities in a cohort of patients with severe obesity submitted to bariatric surgery. Furthermore we investigated the role of DIO2p.Thr92Ala polymorphism in bariatric surgery efficacy.

## Materials and methods

We retrospectively evaluated 182 patients with obesity followed at our Unit of Endocrinology and submitted to bariatric surgery from January 2011 to December 2019. The diagnosis of obesity was based on Body Mass Index (BMI) calculated using the formula: weight in kilograms divided by height in meters squared. According to the Italian Society of Obesity Surgery (SICOB) and International Federation for the Surgery of obesity and metabolic disorders (IFSO) criteria, indications for bariatric surgery are as follows:BMI ≥ 40 kg/m^2^; BMI ≥ 35–40 kg/m^2^ with associated comorbidities; BMI 30–35 kg/m^2^ and type 2 diabetes with poor control despite optimal medical therapy. Different bariatric procedures were performed according to clinical comorbidities of patients: in 19.2% of patients roux-en-Y gastric bypass (RYGB), in 28.1% one anastomosis gastric bypass (OAGB), in 40.6% sleeve gastrectomy (SG) and in 12.1% adjustable gastric banding (AGB). At screening (pre-surgical) and 12 months after surgery (post-surgical), glycaemic control parameters (fasting glucose, insulin and glycated haemoglobin) and cardiometabolic risk biomarkers (waist circumference, systolic and diastolic blood pressure, HDL-cholesterol, LDL-cholesterol, triglycerides) were assessed. The index of insulin resistance, HOMA Index, was calculated as the product of the fasting plasma insulin level (mU/L) and the fasting plasma glucose level (mmol/L), divided by 22.5^22^. Excess Weight (EW) was calculated using the formula [actual body weight − adjusted body weight]. The adjusted body weight was obtained with the formula: [ideal body weight + 0.4 (actual body weight − ideal body weight). For men, the ideal body weight is 50 kg + 2.3 kg for each inch over 5 feet; for women is 45.5 kg + 2.3 kg for each inch over 5 feet^23^. At follow-up visit (6 and 12 months after bariatric surgery) weight and excess weight loss % (EWL%) calculated using the formula: [(final weight − ideal body weight)/ideal body weight] × 100 were evaluated. At follow-up visit cardiometabolic risk biomarkers were also performed.

Thyroid hormones and TSH were evaluated using a chemiluminescent immunometric assay (Access Immunoassay Systems 2006, Beckman Coulter,Milan, Italy). Normal ranges were 3.8–6.3 pmol/L for free T3 (FT3), 7.5–21.1 pmol/L for free thyroxine (FT4), and 2.7–27.7 pmol/L for TSH.

A diagnosis of arterial hypertension was made in the presence of systolic pressure values ≥ 140 mmHg or in the presence of diastolic pressure values ≥ 90 mmHg. Dyslipidemia was defined in the presence of total cholesterol values > 5.17 mmol/L or in the presence of LDL cholesterol values > 3.62 mmol/L. None of the patients were on lipid-lowering therapy. We diagnosed diabetes mellitus in the presence of two findings of fasting glucose ≥ 7 mmol/L, in the presence of two findings of glycated hemoglobin ≥ 47.5 mmol/mol or in the presence of a random glucose finding ≥ 11.11 mmol/mol. Metabolic syndrome was diagnosed in the presence of at least three of: systolic blood pressure ≥ 140 mmHg, diastolic blood pressure ≥ 90 mmHg, triglycerides ≥ 1.69 mmol/L, fasting blood glucose ≥ 5.56 mmol/L, diabetes melllitus, HDL-cholesterol < 1.03 mmol/L in men and < 1.29 mmol/L in women, abdominal circumference > 1.02 m in men and > 0.88 m in women.

A written informed consent was obtained from each patient.

### DNA extraction and DIO2 Thr92Ala analysis

Genomic DNA was extracted from peripheral blood leukocytes using standard procedures^24^ and concentration was assessed with Nanodrop One (Thermo Scientific, Milan, Italy). DIO2 p.Thr92Ala was investigated as described^8^.

### Statistical analysis

Epidemiological data are presented as the mean ± SD and median when needed. The categorical variables were compared between groups using a Chi-squared test or Kruskall Wallis test when needed. Interaction with SNP was tested by Chi-squared test at genotype and allele levels. In addition to basic tests, the association of genotype was evaluated assuming dominant and recessive models. Statistical analysis was performed using the software package SPSS v13.0. A p value < 0.05 was considered statistically significant.

### Ethical approval

All procedure performed in studies involving human participants were in accordance with the ethical standards of the institutional research committee and with the 1964 Helsinki declaration and its later amendments or comparable ethical standards. The study was approved by our local ethical committee “Office of Ethical Affairs, Azienda Ospedaliero-Universitaria Senese”.

### Consent to participate statement

A written informed consent was obtained from each patient.

## Results

### Baseline characteristics and prevalence of DIO2 p.Thr92Ala polymorphism in the study population

Our study population included 135 females (74.2%) and 47 males (25.8%). The mean age was 43 ± 11 years (range 18–68 years), the mean BMI was 45.3 ± 7.0 kg/m^2^ with range 33.9–78.9 kg/ m^2^ (median 44.8 kg/m^2^ and interquartile range 9.1 kg/m^2^) and the mean excess weight (EW) was 40 ± 12.7 kg (range 21.1–99.2 kg). Based on genotype evaluation, 78/182 (42.9%) patients were homozygous wild-type (Thr/Thr), 83/182 (45.6%) were heterozygous (Thr/Ala), and 21/182 (11.5%) were rare homozygous (Ala/Ala). These frequencies were similar to those observed in European and Tuscany populations in particular according with Ensembl Genome Browser Database (ENSG00000211448)^24^ (Table 1).

**Table 1:** Genotype distribution in European, Tuscany and our obese population.

	European (%)	Tuscany (%)	Study population n = 182 (%)
Thr/Thr	45.1	39.3	42.9
Thr/Ala	40.8	49.5	45.6
Ala/Ala	14.1	11.2	11.5

### Anthropometric and metabolic results according to p.Thr92Ala variant

The results of association between the presence/absence of DIO2 p.Thr92Ala and anthropometric characteristics, circulating free thyroid hormones, TSH, lipid profile are shown in Table 2. Age at the time of the first evaluation in our Unit was significantly lower in patients with DIO2 Thr92Ala polymorphism (p = 0.03) even if we considered dominant and recessive model. BMI was similar among the three groups at genotype level but homozygous Ala/Ala patients showed a waist circumference lower than wild type and heterozygous subjects [cm 117.5 ± 13.1 (Ala/Ala) vs. 127.4 ± 17.1 (Thr/Ala) and 124.7 ± 14.8 (Thr/Thr); p = 0.05]. No association at genotype and allele levels was found between the DIO2 p.Thr92Ala and LDL-cholesterol, triglycerides and HOMA IR. EW was near to significance (p = 0.07) being lower for homozygous Ala/Ala (36.1 ± 9.2 kg) compared with wild type (38.7 ± 12.2 kg) and heterozygous (42.1 ± 13.6 kg) subjects. Wild type Thr/Thr patients had greater fasting glucose levels compared with heterozygous and rare homozygous [mmol/L 6.57 ± 3.15 (Thr/Thr) vs. 6.42 ± 2.4 (Thr/Ala) and 5.36 ± 0.61 (Ala/Ala); p = 0.04]. DIO2 Thr92Ala variant was not associated with variation in TSH, FT4 and FT3 values.

**Table 2:** Clinical features according with genotype in 182 obese subjects.

	Thr/Thr (n = 78) Mean ± SD	Thr/Ala (n = 83) Mean ± SD	Ala/Ala (n = 21) Mean ± SD	p value
Age (years)	45.4 ± 11.34	41.1 ± 10.8	44.6 ± 11.5	0.03
Sex (female:male)	61:17	56:27	18/3	0.1
BMI (kg/m^2^)*	43[7.8]	46.1 [10.7]	42 [9.9]	0.1
EW (kg)	38.7 ± 12.2	42.1 ± 13.6	36.1 ± 9.2	0.07
Waist (cm)	124.7 ± 14.8	127.4 ± 17.1	117.5 ± 13.1	0.05
LDL-cholesterol (mmol/L)	2.87 ± 0.79	2.84 ± 0.83	3.15 ± 0.81	0.2
HOMA_IR_*	3.8 [5]	4.6 [5.2]	3.3 [4]	0.3
TSH (pmol/L)*	15.27 [9.02]	27.08 [14.58]	13.19 [8.33]	0.2
fT3 (pmol/L)	5.7 ± 2.5	5.4 ± 0.6	5.2 ± 0.6	0.3
fT4 (pmol/L)	11.2 ± 1.8	11.1 ± 2.1	10.9 ± 1.4	0.8

*Variables not normally distributed expressed in median [interquartile range].

### Prevalence of obesity comorbidities according to p.Thr92Ala variant

Obesity-related comorbidities were evaluated in a subgroup of patients (n = 133) with complete 12 months follow-up. The prevalence of hypertension was 50.4% (67/133), of T2DM was 24% 32/133), of dyslipidemia was 45.9% (61/133), of metabolic syndrome was 51.9% (69/133). As shown in Table 3, the prevalence of comorbidities was not associated with allele distribution except for hypertension that was more frequent in wild-type patients (p = 0.03).

**Table 3:** Prevalence of obesity comorbidities in Thr92Ala subgroups.

	Thr/Thr Prevalence (%)	Thr/Ala Prevalence (%)	Ala/Ala Prevalence (%)	p value
Hypertention	61.4% (35/57)	37.9% (22/58)	55.5% (10/18)	0.03
Diabetes mellitus	22.8% (13/57)	25.9% (15/58)	22.2% (4/18)	0.9
Dyslipidemia	50.9% (29/57)	37.9% (22/58)	55 5% (10/18)	0.2
Metabolic syndrome	56.1% (32/57)	48.2% (28/58)	50% (9/18)	0.6

### DIO2 p.Thr92Ala and weight loss after bariatric surgery

**Figure 1:**
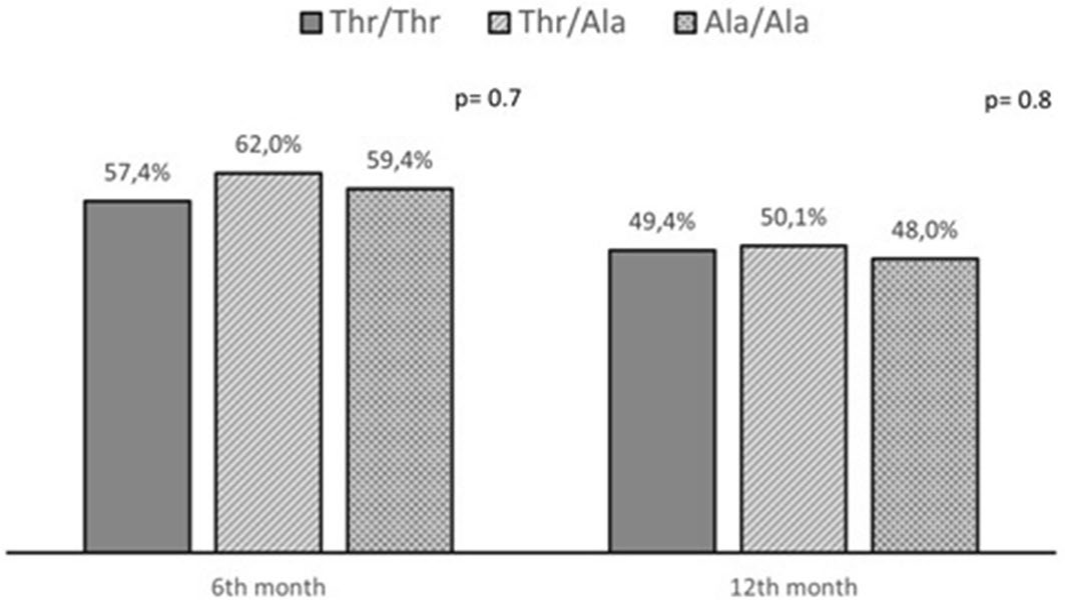
EWL% evaluated at 6 and 12 months of follow-up after bariatric surgery according to genotype (Thr/Thr, Thr/Ala, Ala/Ala), with no statistically significant differences between the three groups (p = 0.7 and p = 0.8).

Weight loss data were available in 150/182 (82.4%) patients at 6 months follow-up and in 133/182 (73%) patients at 12 months of follow up. The mean EWL% at 6 and 12 months was 57.4% and 49.4% in wild type patients, 62% and 50.1% in Thr/Ala group, 59.4% and 48% in Ala/Ala group, respectively, without significant differences (p = 0.7 and p = 0.8, respectively) (shown in Fig. 1).

By separately evaluating the two types of surgery, restrictive (SG and AGB) and malabsorptive (RYGB e OAGB), there were no significant differences in terms of EWL% in the three genotypes (p = 0.7 at 6 months and p = 0.6 at 12 months for restrictive surgery; p = 0.4 at 6 months and p = 0.9 at 12 months for malabsorptive surgery).

### p.Thr92Ala polymorphism and obesity-related comorbidities after bariatric surgery

The rate of remission of comorbidities was evaluated at 12 months follow-up (Table 4). Remission from hypertension occurred in 40% of patients (27/67), without differences between genotypes (p = 0.7). Similarly, remission from diabetes mellitus occurred in 93.7% of patients (30/32) without differences between the *DIO2* genotypes (p = 0.8). Remission from dyslipidemia and metabolic syndrome occurred in 57.4% (35/61) and 81.1% (56/69) of patients respectively, without differences between the *DIO2* genotypes (p = 0.7 and p = 0.7, respectively).

**Table 4:** Remission of comorbidities after bariatric surgery according to p.Thr92Ala subgroups (12th month).

	Thr/Thr Prevalence (%)	Thr/Ala Prevalence (%)	Ala/Ala Prevalence (%)	p value
Hypertention	37.1% (13/35)	40.9% (9/22)	50% (5/10)	0,7
Diabetes mellitus	92.3% (12/13)	93.3% (14/15)	100% (4/4)	0,8
Dyslipidemia	55.1% (16/29)	63.6% (14/22)	50% (5/10)	0,7
Metabolic Syndrome	78.1% (25/32)	85.7% (24/28)	77.7% (7/9)	0,7

## Discussion

Several previous studies suggested an association between p.Thr92Ala polymorphism and hypertension^10^, bipolar disorder^11^, mental retardation^12^, low intelligence quotient (IQ)^13^, recovery from lung injury^14^, osteoarthritis^15^, increased bone turnover^16^, Grave’s and Hashimoto’s diseases^17^. To our knowledge no studies were focused on patients with obesity and, in particular on subjects affected by severe obesity (median of BMI 44.8 kg/m^2^) undergoing to bariatric surgery. We investigated the DIO2p.Thr92Ala polymorphism in relation to obesity and its comorbidities in a group of patients submitted to bariatric surgery. The prevalence of DIO2 p.Thr92Ala polymorphism observed (11.5% Ala/Ala homozygous variant) was similar to that reported in the general European population (14.1% Ala/Ala homozygous variant) and in the Tuscany population (11.2% Ala/Ala homozygous variant)^25^, excluding a possible role of the p.Thr92Ala polymorphism in promoting the onset of severe obesity. Similar results were observed in a relatively large population-based cohort of whites in which DIO2 p.Thr92Ala genotype was evaluated in patients with and without obesity^19^. When a dominant or a recessive model for the penetrance of Ala92 allele were used, no association with obesity was detected^16,18,19^. On the contrary, other published studies reported a significant association between p.Thr92Ala polymorphism and BMI, but only when patients without obesity were evaluated^26^. Moreover a correlation was observed only in patients with both p.Thr92Ala and p.Trp64Arg ADRB3 polymorphisms^4,18^. This interaction was not replicated in studies on the Pima Indians^16^ and in another large cohort of more than 7000 Western Europe subjects^19^. In our study an early-onset of severe obesity was significantly associated with DIO2 polymorphism. In details, the mean age at bariatric surgery was 45.4 ± 11.3 years in wild type patients, 41.1 ± 10.8 years in patients with rare homozygous variant (Ala/Ala) and 44.6 ± 11.5 in heterozygous (Thr/Ala) patients (p = 0.03). These results support a possible role of DIO2 p.Thr92Ala polymorphism in contributing to early-onset of severe obesity, although we do not believe that this is a major determinant for obesity among our study population. To our knowledge no published studies evaluated the association between obesity-related comorbidities and p.Thr92Ala polymorphism. We did not found any association between p.Thr92Ala polymorphism and the presence of obesity-related comorbidities such as diabetes mellitus, liver steatosis, and sleep apnoea syndrome or metabolic syndrome. Published studies in which the association between the DIO2 p.Thr92Ala polymorphism and glycemic control in T2DM patients has been explored, reported contradictory results^2,3,19,27^. Nevertheless, a recent meta-analysis found that people who are homozygous for p.Thr92Ala had 4.8% higher HbA1C levels, suggesting that Ala/Ala homozygosity may be associated with worse glycemic control in T2DM patients^28^. Surprisingly, higher fasting glucose levels and a higher rate of hypertension was found in wild type patients. Gumieniak et al. reported that the Ala allele increases the risk for development of arterial hypertension^10^. These apparently conflicting findings might be partially explained by differences in the studied population. In Gumieniak’s study antihypertensive medications were stopped 2–4 weeks before the study; the cut-offs were different (diastolic blood pressure of 100 mmHg off antihypertensive medications, 90 mmHg while taking 1 or 2 medication); subjects with hypertension requiring 4 medications were excluded. In our study no exclusion criteria for hypertension were used, the diagnosis of arterial hypertension was made in the presence of systolic pressure values ≥ 140 mmHg or in the presence of diastolic pressure values ≥ 90 mmHg. Furthermore, in the Gumieniak’s study the patients were overweight (mean BMI 27.7 ± 4.2 kg/m2) while our study population included only patients with severe obesity. To explain the discrepancies between our results and published data, we might speculate that the contribution made by severe obesity in determining associate comorbidities was greater than that of polymorphism.

Bariatric surgery is currently the most effective tool in determining an important and lasting weight loss in patients with obesity; in addition a high rate of remission of the main obesity-related comorbidities has been reported after bariatric surgery^29–35^. In our study, bariatric surgery was effective, in terms of weight loss (EWL%) in all patients, regardless the genotype. Moreover, bariatric surgery promotes remission or a significant improvement of obesity related comorbidities without significant differences between the three groups.

## Conclusion

Based on the present investigations, performed in relatively large samples of patients, we conclude that the DIO2p.Thr92Ala polymorphism does not affect the severity of obesity and its complications, but it seems to determine an earlier onset of complicated obesity. Furthermore, the presence of polymorphism does not impact on the response to surgical treatment, both in terms of weight loss and remission of comorbidities.

## Data Availability

The data that support the findings of this study are not publicly available due to their containing information that could compromise the privacy of research participants but are available from corresponding author [M.G.C] upon reasonable request.
